# Re-establishment of efficacy of tofacitinib, an oral JAK inhibitor, after temporary discontinuation in patients with rheumatoid arthritis

**DOI:** 10.1007/s10067-020-04956-1

**Published:** 2020-02-12

**Authors:** Jeffrey Kaine, John Tesser, Liza Takiya, Ryan DeMasi, Lisy Wang, Mark Snyder, Koshika Soma, Haiyun Fan, Vara Bandi, Jürgen Wollenhaupt

**Affiliations:** 1 Independent Healthcare Associates Inc, Cullowhee, NC USA; 2 Arizona Arthritis & Rheumatology Associates, Glendale, AZ USA; 3 Pfizer Inc, Collegeville, PA USA; 4 Pfizer Inc, Groton, CT USA; 5 Eliassen Group Inc, New London, CT USA; 6 Rheumatology im Struenseehaus, Hamburg, Germany

**Keywords:** Dose interruption, Efficacy, Rheumatoid arthritis, Safety, Tofacitinib

## Abstract

**Introduction/objective:**

Tofacitinib is an oral Janus kinase inhibitor for the treatment of rheumatoid arthritis (RA). This post-hoc analysis evaluated the effect of temporary discontinuation and reinitiation of tofacitinib on disease control in patients with RA in the vaccine sub-study of the long-term extension (LTE) study ORAL Sequel (NCT00413699).

**Methods:**

The sub-study of ORAL Sequel was a randomized, parallel-group, open-label study. Patients who received tofacitinib 10 mg twice daily for ≥ 3 months in ORAL Sequel were randomized to receive continuous (tofacitinib monotherapy/with methotrexate) or interrupted (tofacitinib withdrawn for 2 weeks post-randomization then reinitiated as monotherapy/with methotrexate) treatment. Efficacy assessments included ACR20/50/70 response rates, change from baseline (∆) in C-reactive protein (CRP), Health Assessment Questionnaire-Disability Index (HAQ-DI), Disease Activity Score in 28 joints, erythrocyte sedimentation rate (DAS28-4 [ESR]), Clinical Disease Activity Index (CDAI), Patient Global Assessment of arthritis (PtGA), Pain (Visual Analog Scale [VAS]), and Physician Global Assessment of arthritis (PGA). Safety was assessed throughout.

**Results:**

The sub-study included 99 patients each in the continuous and interrupted treatment groups. ACR20/50 response rates, ∆CRP, ∆HAQ-DI (day 15), ∆DAS28-4 (ESR), ∆CDAI, ∆PtGA, ∆Pain (VAS), and ∆PGA were significantly worse in interrupted vs continuous patients during dose interruption, but were generally similar to pre-interruption/continuous treatment levels 28 days post-reinitiation. A numerically higher proportion of interrupted patients reported adverse events (49.5%) vs continuous patients (35.4%).

**Conclusions:**

Tofacitinib efficacy can be re-established after temporary withdrawal and reinitiation. The safety profile of patients who temporarily discontinued tofacitinib in the sub-study was consistent with previous tofacitinib LTE studies over 9 years.

**Clinical trial registration number:**

NCT00413699

**Table:** 

**Key Points** • *In this sub-study of the long-term extension (LTE) study, ORAL Sequel, the efficacy of tofacitinib was re-established after temporary withdrawal (2 weeks) and reinitation of treatment in patients with RA.* • *Patients with RA who temporarily discontinued tofacitinib had similar safety events to those reported in previous LTE studies.* • *The results of this sub-study were consistent with a post-hoc analysis of pooled data from two LTE studies, ORAL Sequel and A3921041, which assessed the efficacy of tofacitinib following a treatment discontinuation period of 14–30 days.*

## Introduction

Rheumatoid arthritis (RA) is an autoimmune disease affecting approximately 0.24% of the population worldwide [1]. Characterized by systemic inflammation, persistent synovitis and, ultimately, joint destruction [2], it can lead to reduced productivity and impaired health-related quality of life [3].

As a chronic disease, RA requires long-term treatment; however, there may be situations when medication is temporarily discontinued, such as when patients are managing comorbidities (e.g., undergoing surgery or having treatment for infections), drug interactions, or laboratory abnormalities. Previous studies of biologic disease-modifying antirheumatic drugs (bDMARDs) have shown that the temporary discontinuation of treatment was associated with a flare in disease and loss of remission, which was reversed after treatment reinitiation [4–8].

Tofacitinib is an oral Janus kinase inhibitor for the treatment of RA. The efficacy and safety of tofacitinib 5 and 10 mg twice daily (BID) administered as monotherapy or in combination with conventional synthetic (cs)DMARDs, mainly methotrexate (MTX), in patients with moderately to severely active RA, have been demonstrated in Phase 2 [9–13], Phase 3 [14–19], and Phase 3b/4 [20] randomized controlled trials (RCTs) of up to 24-months' duration, and in long-term extension (LTE) studies with up to 114 months of observation [21–23].

The tofacitinib LTE study ORAL Sequel (NCT00413699) [21, 23] included a sub-study to assess immune response following the administration of influenza and pneumococcal vaccines in patients with RA receiving tofacitinib, who were randomized to receive a continuous or interrupted tofacitinib treatment regimen [24]. The temporary discontinuation of tofacitinib did not appear to affect the responses to either vaccine [24]. Here, we assess the efficacy and safety of tofacitinib during and after 2 full weeks of temporary discontinuation and reinitiation in patients with RA who participated in the sub-study of ORAL Sequel.

## Methods

### Study design

ORAL Sequel (A3921024; NCT00413699) was a global, multicenter, open-label LTE study [21, 23]. Patients were eligible if they were aged ≥ 18 years, with a diagnosis of RA based on the American College of Rheumatology (ACR) 1987 Revised Criteria, and had participated in a prior Phase 1, 2, or 3 qualifying index study of tofacitinib. The majority of patients who had participated in a Phase 2 qualifying study initiated treatment in ORAL Sequel with tofacitinib 5 mg BID, whereas the majority of patients who had participated in a Phase 3 qualifying study initiated treatment with tofacitinib 10 mg BID, with the exception of patients from China and Japan who initiated treatment with tofacitinib 5 mg BID as per the protocol. The tofacitinib dose could be reduced from 10 to 5 mg BID for safety reasons, or increased from 5 to 10 mg BID for reasons of inadequate response. Dose adjustments were at the discretion of the investigator and could be temporary or last the duration of the study. Permitted concomitant RA medications included MTX, leflunomide, sulfasalazine, antimalarials, auranofin, injectable gold preparations, nonsteroidal anti-inflammatory drugs, and glucocorticoids (at approved doses); these were subject to dose adjustments, tapering, and discontinuation at the discretion of the investigator.

Following an amendment to the ORAL Sequel protocol, a sub-study was performed to assess immune response in patients receiving tofacitinib following administration of pneumococcal and influenza vaccines [24]. Patients in this randomized, parallel-group open-label study must have received tofacitinib 10 mg BID for ≥ 3 months in ORAL Sequel. Those who were receiving MTX must have taken this continuously for ≥ 4 months, and have been at a stable weekly dose (7.5–25 mg/week) for ≥ 6 weeks, before randomization. Doses of < 7.5 mg/week were allowed only in cases of intolerance/toxicity, or where higher doses had the possibility to violate the local label. Patients receiving other concomitant RA medications were required to receive stable doses of such medications for ≥ 4 weeks prior to randomization.

**Fig. 1:**
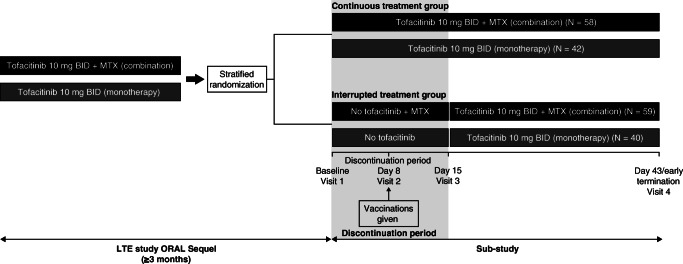
Schematic of the sub-study of ORAL Sequel. *BID* twice daily; *LTE* long-term extension; *MTX* methotrexate

Patients in the sub-study were randomized 1:1 to receive continuous or interrupted treatment (Fig. 1), with stratification by current use of background MTX. The continuous treatment sequence included patients who received tofacitinib 10 mg BID as monotherapy or with MTX. This treatment arm was included to confirm that patients who stopped and restarted treatment had comparable disease activity levels post-reinitiation with those who continued treatment. In the interrupted treatment sequence, tofacitinib 10 mg BID was withdrawn for 2 weeks post-randomization (days 1 to 15), and was subsequently reinitiated as monotherapy or with MTX (as per the original stratification) at day 15 (visit 3). Pneumococcal and influenza vaccines were administered to all patients on day 8 (visit 2). Assessments performed on visit 1 were considered as baseline for the sub-study.

The study was conducted in accordance with the Declaration of Helsinki and the International Conference on Harmonization Good Clinical Practice Guidelines and was approved by the Institutional Review Boards and/or Independent Ethics Committees at each investigational center. All patients provided written informed consent.

### Outcomes and statistical analyses

The primary assessment in this post-hoc analysis was the efficacy and safety of tofacitinib after a 2-week temporary discontinuation period followed by treatment reinitiation in patients who participated in the sub-study. Efficacy endpoints were assessed at visit 1 (baseline), visit 2 (day 8; vaccination), visit 3 (day 15; end of withdrawal period; tofacitinib reinitiation for interrupted group), and visit 4 (day 43 [28 days post-reinitiation] or at early termination), and included ACR20, ACR50, and ACR70 response rates, which were defined as ≥ 20%, ≥ 50%, and ≥ 70% improvement, respectively, from the baseline of the LTE study, in tender joint count and swollen joint count, and in ≥ 3 of the other 5 ACR components. The LTE study baseline was the baseline of the index study for patients enrolling into the LTE study ≤ 14 days from last tofacitinib index study dose, or baseline of ORAL Sequel for patients enrolling into the LTE study > 14 days from last tofacitinib index study dose. Further outcomes evaluated were changes from the sub-study baseline (∆) in C-reactive protein (CRP), Health Assessment Questionnaire-Disability Index (HAQ-DI), Disease Activity Score in 28 joints, erythrocyte sedimentation rate (DAS28-4 [ESR]), Clinical Disease Activity Index (CDAI), Patient Global Assessment of arthritis (PtGA), Pain Visual Analog Scale (VAS) score, and Physician Global Assessment of arthritis (PGA). Safety data were assessed throughout, and included adverse events (AEs), serious AEs (SAEs), and discontinuations due to AEs.

The efficacy analyses included patients who were randomized to the sub-study and were treated with tofacitinib monotherapy or combination therapy. Patients from the continuous treatment group who had received tofacitinib for ≥ 1 day, and patients from the interrupted treatment group who were withdrawn from tofacitinib for ≥ 1 day, were considered as having been treated and were included in the efficacy analyses.

A mixed-effects model with repeated measures was used to evaluate the treatment effect for continuous endpoints such as ∆CRP, ∆HAQ-DI, ∆DAS28-4 (ESR), ∆CDAI, ∆PtGA, ∆Pain (VAS), and ∆PGA at visits 2, 3, and 4 (days 8, 15, and 43, respectively). This method was selected to maximize the use of all data and thus to increase the validity and efficiency of the model; furthermore, the mixed-effects model can handle missing values if random, as it was assumed to be in most cases. Background MTX use, region, and baseline values were included as covariates, with treatment, visit, and treatment-by-visit interaction as fixed factors. The 95% confidence interval (CI) of the treatment difference (continuous treatment group–interrupted treatment group), and the *P* value for the treatment comparison, were calculated within the model. For binary endpoints of ACR response rates, normal approximation for binomial proportions was used to assess the treatment effect. These analyses were exploratory in nature; no multiplicity adjustment was made for comparisons.

The safety analyses included patients who received ≥ 1 tofacitinib dose or who discontinued tofacitinib during the sub-study of ORAL Sequel.

## Results

### Patients

Overall, 199 patients in the sub-study were randomized to receive study treatment (continuous treatment *N* = 100; interrupted treatment *N* = 99); 198 were treated with tofacitinib for ≥ 1 day or withdrawn from tofacitinib for ≥ 1 day. There were 16 patients with protocol deviations who were not evaluable in the immunogenicity analysis but were included in this post-hoc analysis. Of the 100 patients randomized to continuous treatment, 99 received continuous treatment with tofacitinib, and 58 (58.6%) of these patients received concomitant MTX (mean [standard deviation (SD)] baseline dose 14.1 [4.1] mg/week). Of the 99 patients randomized to interrupted treatment, all received interrupted treatment with tofacitinib and 59 (59.6%) received concomitant MTX (mean [SD] baseline dose 15.9 [3.9] mg/week).

Baseline demographics and disease characteristics are presented in Table 1. The continuous and interrupted treatment groups were comparable in terms of age, sex, and race. Baseline values for CRP, HAQ-DI, DAS28-4 (ESR), CDAI, PtGA, Pain (VAS), and PGA appeared generally similar between the continuous and interrupted treatment groups at the start of the sub-study, although CRP values showed a high degree of variability in the interrupted treatment group.

**Table 1:** Demographics and disease characteristics of patients in the ORAL Sequel sub-study^a^

	Tofacitinib 10 mg BID Continuous (*N* = 99)	Tofacitinib 10 mg BID Interrupted (*N* = 99)
Age (years), mean (SD)	55.0 (11.3)	53.9 (9.2)
Sex, *n* (%)		
Male	15 (15.2)	13 (13.1)
Female	84 (84.8)	86 (86.9)
Race, *n* (%)		
White	81 (81.8)	83 (83.8)
Black	3 (3.0)	1 (1.0)
Asian	13 (13.1)	14 (14.1)
Other	2 (2.0)	1 (1.0)
BMI (kg/m^2^), mean (SD)	27.3 (6.1)	28.2 (6.9)
CRP (mg/L), mean (SD)	2.5 (3.0)	4.1 (10.2)
HAQ-DI, mean (SD)	0.9 (0.7)	1.0 (0.7)
DAS28–4 (ESR), mean (SD)	3.6 (1.3)	3.7 (1.3)
CDAI, mean (SD)	10.5 (9.5)	11.9 (11.6)
PtGA (mm), mean (SD)	30.6 (21.3)	33.7 (22.9)
Pain (VAS) (mm), mean (SD)	28.5 (22.2)	32.2 (23.1)
PGA (mm), mean (SD)	16.8 (13.2)	18.9 (16.3)
Mean MTX dose (mg/week), mean (SD)	14.1 (4.1) [*n* = 58]	15.9 (3.9) [*n* = 59]

^a^At the sub-study baseline (prior to interruption). *BID* twice daily; *BMI* body mass index; *CDAI* Clinical Disease Activity Index; *CRP* C-reactive protein; *DAS28-4 (ESR)* Disease Activity Score in 28 joints, erythrocyte sedimentation rate; *HAQ-DI* Health Assessment Questionnaire-Disability Index; *MTX* methotrexate; *PGA* Physician Global Assessment of arthritis; *PtGA* Patient Global Assessment of arthritis; *SD* standard deviation; *VAS* visual analog scale

### Efficacy assessments

**Fig. 2:**
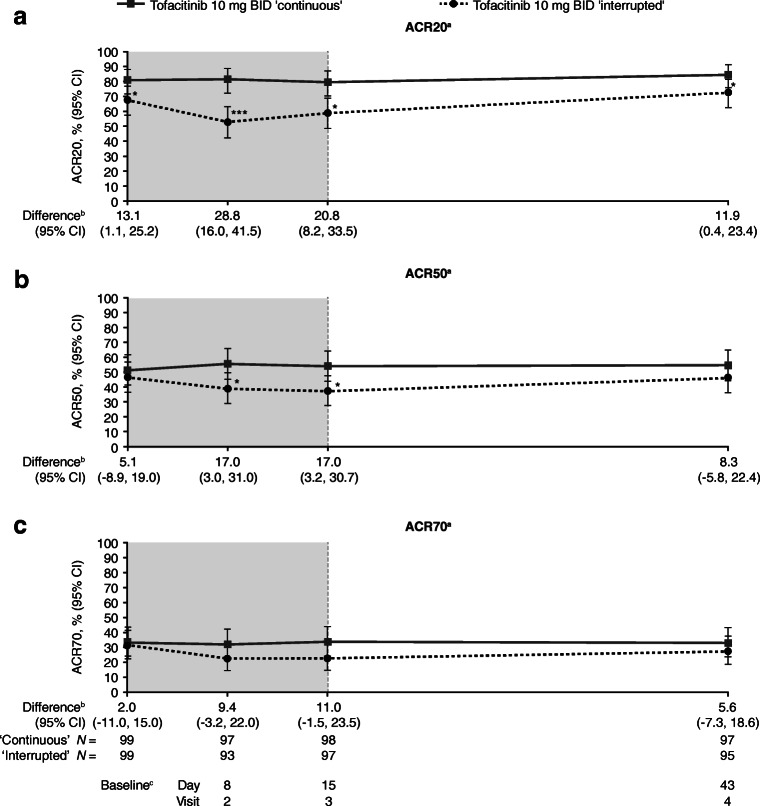
**a** ACR20 response rates, **b** ACR50 response rates, and **c** ACR70 response rates over time during the sub-study of ORAL Sequel. *p < 0.05; **p < 0.001; ***p < 0.0001 for interrupted vs continuous treatment. Shaded area indicates the dose interruption period. ^a^The ACR response rate at each visit is the proportion of patients achieving improvement from the baseline of the index study for patients enrolling into the LTE study ≤14 days from last tofacitinib dose in the index study or baseline of ORAL Sequel for patients enrolling into the LTE study >14 days from last tofacitinib dose in the index study ^b^ACR response rate of continuous treatment group – response rate of interrupted treatment group ^c^Baseline visit of sub-study. *ACR* American College of Rheumatology; *BID* twice daily; *CI* confidence interval; *LTE* long-term extension

At the sub-study baseline, the proportion of patients who achieved ACR20 response (95% CI) was significantly higher in patients receiving continuous tofacitinib treatment (80.8% [71.7, 88.0] vs interrupted treatment (67.7% [57.5, 76.7]; difference 13.1% [1.1, 25.2]; *p* < 0.05) (Fig. 2a). The difference between the two groups increased to 28.8% (16.0, 41.5; *p* < 0.0001) at day 8, as although the ACR20 response rate remained fairly constant for patients receiving continuous treatment (81.4% [72.3, 88.6]), it decreased in patients with interrupted treatment (52.7% [42.1, 63.1]). By day 43 (28 days after treatment reinitiation), the ACR20 response rate in the interrupted treatment group had increased to 72.6% (62.5, 81.3), but was still significantly different from the continuous treatment group (84.5% [75.8, 91.1]; difference 11.9 [0.4, 23.4]; *p* < 0.05).

ACR50 response rates (95% CI) were similar at sub-study baseline between patients who received continuous (51.5% [41.3, 61.7]) vs interrupted treatment (46.5% [36.4, 56.8]; difference 5.1% [− 8.9, 19.0]) (Fig. 2b). The ACR50 response rates remained fairly constant for patients receiving continuous treatment (day 8 55.7% [45.2, 65.8]; day 15 54.1% [43.7, 64.2]); day 43 54.6% [44.2, 64.8]); however, the ACR50 responses in the interrupted treatment group decreased slightly and were significantly different from the continuous treatment group at day 8 (38.7% [28.8, 49.4]; difference 17.0% [3.0, 31.0]; *p* < 0.05) and day 15 (37.1% [27.5, 47.5]; difference 17.0% [3.2, 30.7]; *p* < 0.05). By day 43 (28 days after treatment reinitiation), the ACR50 response rate had increased (46.3% [36.0, 56.9]) and was not significantly different from that of the continuous treatment group (difference 8.3% [− 5.8, 22.4]).

ACR70 response rates (95% CI) in the continuous treatment group remained constant throughout the study (sub-study baseline 33.3% [24.2, 43.5]; day 8 32.0% [22.9, 42.2]; day 15 33.7% [24.4, 43.9]; day 43 33.0% [23.8, 43.3]) (Fig. 2c). ACR70 response rates in the interrupted treatment group were numerically lower but were not significantly different from the continuous treatment group throughout the study (sub-study baseline 31.3% [22.4, 41.4]; day 8 22.6% [14.6, 32.4]; day 15 22.7% [14.8, 32.3]; day 43 27.4% [18.7, 37.5]).

**Fig. 3:**
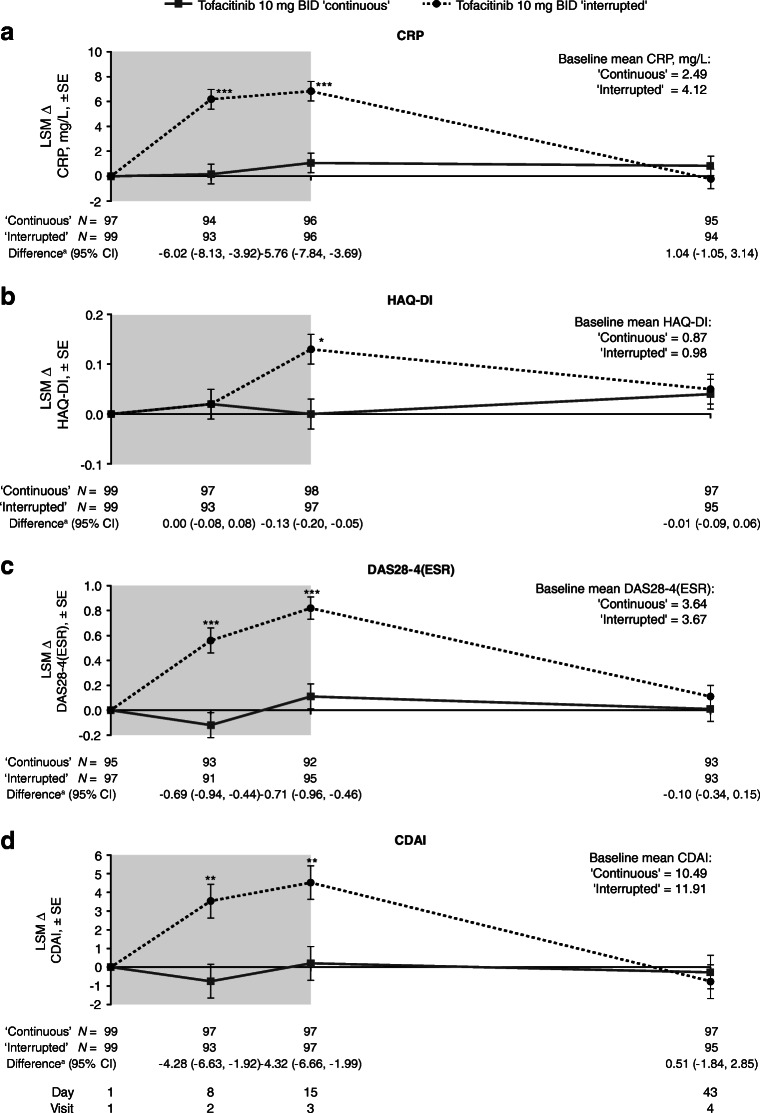
LSM changes from baseline in **a** CRP levels, **b** HAQ-DI, **c** DAS28-4 (ESR), and **d** CDAI over time during the sub-study of ORAL Sequel. *p < 0.05; **p < 0.001; ***p < 0.0001 for interrupted vs continuous treatment. Shaded area indicates the dose interruption period. Baseline was defined as Visit 1 of the sub-study. ^a^LSM Δ of continuous treatment group – LSM Δ of interrupted treatment group. *Δ* change from baseline; *BID* twice daily; *CDAI* Clinical Disease Activity Index; *CI* confidence interval;* CRP* C-reactive protein; *DAS28-4 (ESR)* Disease Activity Score in 28 joints, erythrocyte sedimentation rate; *HAQ-DI* Health Assessment Questionnaire-Disability Index; *LSM* least squares mean; *SE* standard error

**Fig. 4:**
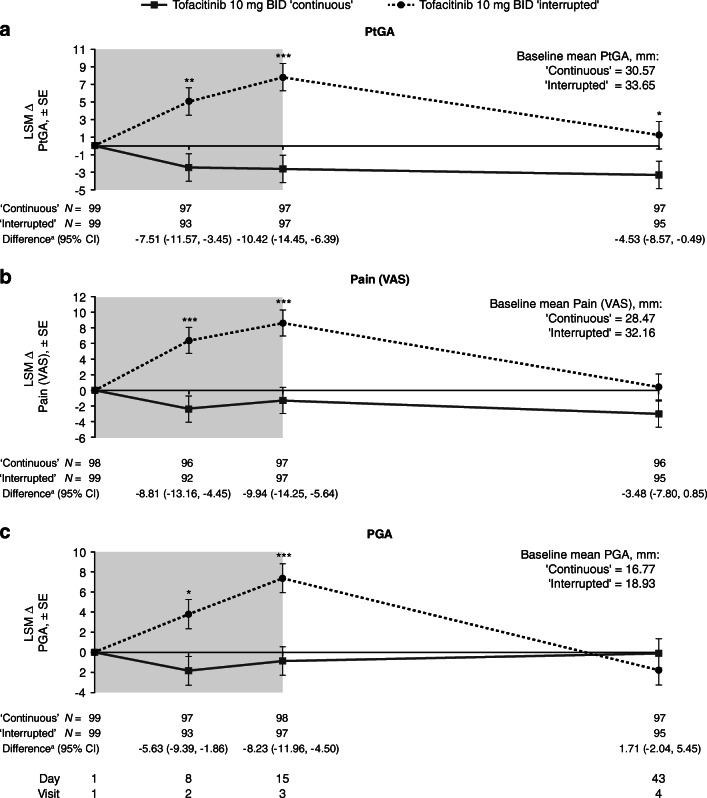
LSM changes from baseline in **a** PtGA, **b** Pain (VAS), and **c** PGA over time during the sub-study of ORAL Sequel. *p < 0.05; **p < 0.001; ***p < 0.0001 for interrupted vs continuous treatment. Shaded area indicates the dose interruption period. Baseline was defined as Visit 1 of the sub-study. ^a^LSM Δ of continuous treatment group – LSM Δ of interrupted treatment group. *Δ* change from baseline; *BID* twice daily; *CI* confidence interval; *LSM* least squares mean; *PGA* Physician Global Assessment of arthritis, *PtGA* Patient Global Assessment of arthritis; *SE* standard error; *VAS* visual analog scale

The increases from baseline in CRP, HAQ-DI, DAS28-4 (ESR), and CDAI were generally significantly greater during treatment interruption vs the continuous treatment group; however, at day 43, changes from baseline in these outcomes were similar to the sub-study baseline level, and there was little change vs the continuous treatment group (Fig. 3). The continuous treatment group showed minimal changes from baseline in these outcomes up to day 43. Unlike CRP, DAS28-4 (ESR), and CDAI, differences in the increases from baseline in HAQ-DI in the two treatment groups were very close at day 8 (1 week post-treatment withdrawal), although they were significantly different at day 15 (2 weeks post-treatment withdrawal) (Fig. 3). Similar trends were also observed in ∆PtGA, ∆Pain (VAS), and ∆PGA, with significant differences between the two groups observed during days 8 and 15 (Fig. 4). At day 43, values were similar to the sub-study baseline level, although the difference between treatments for ∆PtGA was still significant, likely due to the decrease in PtGA observed in the continuous treatment group throughout the sub-study.

### Safety assessments

Overall, a numerically higher proportion of patients reporting AEs (all causality and treatment-related) were observed in the interrupted vs continuous treatment group of the sub-study (Table 2). The most frequent treatment-emergent AEs (all causality) by Medical Dictionary for Regulatory Activities (MedDRA) preferred term across both treatment groups were bronchitis and upper respiratory tract infection, followed by vaccination-related immunization reaction, myalgia, and rash (Table 2).

**Table 2:** Treatment-emergent AEs during the sub-study of ORAL Sequel

	All causalities		Treatment-related	
	Tofacitinib 10 mg BID Continuous (*N* = 99)	Tofacitinib 10 mg BID Interrupted (*N* = 99)	Tofacitinib 10 mg BID Continuous (*N* = 99)	Tofacitinib 10 mg BID Interrupted (*N* = 99)
Number of AEs	50	95	5	14
Patients with AEs, *n* (%)	35 (35.4)	49 (49.5)	4 (4.0)	13 (13.1)
Patients with SAEs, *n* (%)	3 (3.0)	3 (3.0)	0	1 (1.0)
Patients with AEs leading to discontinuation, *n* (%)	0	2 (2.0)	0	1 (1.0)
Most frequent^a^ AEs by preferred term, *n* (%)				
Immunization reaction	1 (1.0)	4 (4.0)	0	0
Bronchitis	2 (2.0)	4 (4.0)	0	1 (1.0)
Upper respiratory tract infection	3 (3.0)	3 (3.0)	1 (1.0)	0
Myalgia	1 (1.0)	4 (4.0)	1 (1.0)	0
Rash	0	5 (5.1)	0	1 (1.0)

^a^Occurring in ≥ 5% of patients (all causalities) across both treatment groups*. AE* adverse event; *BID* twice daily; *SAE* serious adverse event

SAEs occurred in 3 patients in each treatment group; these were cataract, squamous cell carcinoma of the skin, and pharyngeal hemorrhage in the continuous treatment group; and colitis, atrial flutter, and lymph node tuberculosis (considered by the investigator to be treatment-related) in the interrupted treatment group.

Two patients in the interrupted treatment group discontinued treatment due to AEs of colitis and lymph node tuberculosis. One patient in the interrupted treatment group reported an AE of “aggravation of rheumatoid arthritis”; however, this patient did not permanently discontinue treatment due to the AE, and no patients withdrew from the study due to disease flare.

## Discussion

In this analysis, patients who participated in the sub-study of the LTE study ORAL Sequel showed increased disease activity during treatment interruption. Indeed, the ∆DAS28-4 (ESR) in the interrupted treatment group was > 0.6 (the measurement error) [25] at day 15, indicating a relevant increase in DAS28-4 (ESR) when tofacitinib was interrupted. ACR20/50 response rates, ΔCRP levels, ΔHAQ-DI, ΔCDAI, ΔPtGA, ΔPain (VAS), and ΔPGA scores also significantly worsened during treatment interruption vs the continuous treatment group, with physical function (measured by HAQ-DI) appearing to worsen more slowly than the other outcomes. Changes in clinical outcomes can be discordant with patient-reported outcomes [26]; therefore, physicians must be cautious when considering temporary treatment discontinuation; it is possible that disease control might be achieved upon reinitiation, but patients may not always experience the same level of improvement within the same timeframe. However, within 1 month of treatment reinitiation, all efficacy outcomes, except for ACR20 response rate and ΔPtGA, were similar to those before discontinuation. Compared with patients who received continuous treatment, there were also no significant changes in efficacy outcomes (with the exception of ACR20 response rate and ΔPtGA) within 1 month of treatment reinitiation for patients with treatment interruption. It should be noted that ACR20 response rates at the LTE study baseline were significantly higher in patients in the continuous vs interrupted treatment group, which may have skewed the response rates throughout the study; however, other efficacy outcomes, which were measured at the sub-study baseline, were generally similar between the two groups. A numerically higher proportion of patients experienced AEs in the interrupted vs continuous treatment group. The profile of AEs and SAEs, in terms of events reported, was similar to those reported previously in the LTE studies over 9 years [21, 23].

The results of the sub-study were in line with a post-hoc analysis of pooled data from two LTE studies, ORAL Sequel and A3921041, which was performed to confirm the findings from the sub-study and to further assess the efficacy of tofacitinib following a treatment discontinuation period of 14–30 days, which may better reflect real-world circumstances [27]. Similar efficacy responses were observed at pre- and post-interruption visits, suggesting that there was no loss of efficacy after reinitiation of tofacitinib following temporary discontinuation.

Previous studies of other RA therapies have also shown worsening of disease activity upon treatment discontinuation, followed by subsequent regaining of disease control after treatment reinitiation, although these have been assessed over a longer time period. A review of MTX and bDMARDs in non-randomized trials as well as RCTs reported that 35–87% of patients with RA relapsed 1 year after discontinuing treatment in RCTs, with remission and low disease activity (LDA) generally re-established by reinitiation of the previous treatment [28]. In a previous study of patients who had achieved remission or LDA with tumor necrosis factor inhibitors (TNFi) and who had subsequently discontinued treatment, flares were reported in 40.1% and 51.2% of patients within 6 and 12 months, respectively [8]; studies of specific TNFi have shown rates of flares to be 28.4% within 1 year (mean duration 6.4 months) for infliximab and 87.0% within 48 weeks (median time to relapse 6 weeks) for etanercept [4, 5]. Most patients who restarted TNFi treatment after a flare regained disease control [4, 5, 8]. Similarly, a non-randomized study of various TNFi, including adalimumab, etanercept, and infliximab, found that out of 20 patients evaluated, who had achieved remission, 8 (40%) relapsed within 3 months, and 15 (75%) relapsed within 12 months of discontinuing treatment (mean time to relapse 14.7 weeks). All regained remission after reinitiating the same treatment [6]. A Phase 3 RCT, ACT-RAY, has also shown that the discontinuation of tocilizumab in patients with sustained remission resulted in the relapse of 84.0% of patients, with 82.5% (tocilizumab with MTX) or 88.5% (tocilizumab with placebo) experiencing flares within 52 weeks. Rapid improvements in disease activity (measured by DAS28-[ESR]) were observed after tocilizumab reinitiation [7]. Additionally, in the Phase 3b ALLOW study, patients experienced a small increase in disease activity (measured by DAS28-[CRP]) and slight worsening of physical function (measured by HAQ-DI) upon withdrawal of subcutaneous abatacept for 12 weeks; these showed improvement within 1 month of treatment reinitiation [29].

In this sub-study, patients did have a significant increase in disease activity after discontinuing tofacitinib. These patients appeared to experience flares sooner (within 2 weeks) vs the bDMARDs in the aforementioned studies [4–7]. This may be a function of the half-life of the drugs, as tofacitinib immediate-release formulation has a relatively short half-life (~ 3 h) [30] and must be dosed more frequently (BID) than the commonly used bDMARDs (anakinra has a half-life of 4–6 h and is dosed daily; several bDMARDs, including the TNFi, have half-lives ranging from 4 to ~ 14 days [31]).

The sub-study showed that the temporary withdrawal of tofacitinib leads to significant worsening of disease control, but that disease control is regained after treatment reinitiation, suggesting that tofacitinib is an appropriate treatment to be reinitiated following disease activity flares during treatment interruption. Nevertheless, several limitations must be considered when interpreting these results. The sub-study was the only study in the tofacitinib clinical trial program where there was a mandated, randomized, and systematically monitored temporary discontinuation and reinitiation of tofacitinib; however, it was not designed to evaluate the withdrawal effect of tofacitinib. The sample size was also limited by the study design. Moreover, following treatment reinitiation, the follow-up period was relatively short (28 days). As such, further studies with more participants and a longer follow-up period may provide valuable insights into whether there are any long-term impacts of temporary tofacitinib withdrawal. Furthermore, the dose of tofacitinib used was 10 mg BID; however, the approved dose in most countries for the treatment of moderately to severely active RA is 5 mg BID. Additionally, all patients in the sub-study received vaccinations at day 8, which may have affected both the treatment response and the occurrence of AEs. A final limitation is that the patients in this analysis were part of the ORAL Sequel study population, and therefore had previously tolerated and responded to tofacitinib in the index studies.

In summary, the results of this analysis showed that disease outcomes worsened during the temporary discontinuation of tofacitinib, and that the efficacy of tofacitinib can be re-established after the temporary withdrawal and reinitiation of the drug. The profiles of treatment-emergent AEs were consistent with AEs previously reported with long-term tofacitinib treatment [21, 23].
